# Pharmacokinetic Profiling Evaluation of *Ruditapes philippinarum* Polysaccharide

**DOI:** 10.3390/md24080278

**Published:** 2026-08-11

**Authors:** Meng-Yue Liu, Dong-Li Yin, Jia Yu, Wei-Xia Wang, Sheng-Can Zou, Shuang Zhao, Chun-Ze Zou, Fei Li, Yu-Xi Wei

**Affiliations:** 1 College of Life Sciences, Qingdao University, Qingdao 266071, China; 2 Qingdao Yihai Industry Holdings Co., Ltd., Qingdao 266105, China; 3 Donald Bren School of Information and Computer Sciences, University of California, Irvine, CA 92697, USA

**Keywords:** *Ruditapes philippinarum*, polysaccharides, pharmacokinetics, near-infrared fluorescence imaging

## Abstract

Oral administration represents the primary route for administering polysaccharides. Pharmacokinetics serves as a critical bridge between the administration of polysaccharides and the manifestation of their bioactivities. However, the pharmacokinetic behavior of polysaccharides remains poorly understood. This study aimed to investigate the *in vivo* pharmacokinetics of an orally administered *Ruditapes philippinarum* polysaccharide (ERPP), including its plasma concentration–time profile, tissue distribution, and excretion. Moreover, a sensitive quantitative method was developed using 5-DTAF as a fluorescent probe to quantify ERPP levels in rat plasma and tissues. The results revealed that ERPP was absorbed in the gut (T_max_ = 3 h), reaching a peak concentration (C_max_) of 22.7 ± 2.7 mg/L. Subsequently, it predominantly accumulated in the kidneys, liver, and lungs. Complementarily, near-infrared fluorescence (NIR) imaging provided real-time qualitative visualization of ERPP-Cy5.5 tissue distribution, corroborating the organ-level accumulation pattern observed in the quantitative study. Excretion studies over a 24 h observation window indicated that ERPP was primarily excreted via feces, with 45.7 ± 4.4% of the recovered dose within this period; the complete excretion profile warrants further investigation at extended time points. These findings provide pharmacokinetic insights into the *in vivo* fate of marine polysaccharides and support further biological evaluation.

## 1. Introduction

*Ruditapes philippinarum* is one of the most economically important shellfish species in Chinese and global traditional fisheries. It has been widely cultivated in the coastal areas of China, Korea and Southeast Asia, accounting for approximately 90% of the total global production [1,2,3]. Apart from being a nutritious seafood, *R. philippinarum* also holds a well-established role in traditional Chinese medicine. Historically, it has been utilized for its supposed anti-inflammatory and expectorant properties [4,5]. Studies in modern nutrition have shown that *R. philippinarum* is a rich source of bioactive compounds, such as polypeptides and polysaccharides, both of which have been demonstrated to exhibit significant biological activities [5,6]. Numerous previous studies have confirmed that *R. philippinarum* polypeptides possessed notable flavor-enhancing and anti-inflammatory properties [7,8,9].

In recent years, with the advancements in analytical technologies (particularly in the fields of bioactivity screening and omics), an increasing number of experimental studies have focused on the biological functions of polysaccharides [10,11]. These investigations have revealed that *R. philippinarum* polysaccharides have a variety of pharmacological activities, including immunomodulation, anticoagulation, antitumor effect, and antioxidant capacity [4,5,12]. Our previous studies have indicated that *R. philippinarum* polysaccharides can induce tumor cell apoptosis through activation of the mitochondrial pathway, suppress the Wnt/β-catenin signaling pathway, and trigger cell cycle arrest at the *G0/G1* phase, thereby contributing to their antitumor efficacy [13]. Due to their diverse bioactivities, favorable safety profile, and promising therapeutic potential, *R. philippinarum* polysaccharides have attracted increasing interest for applications in functional foods and pharmaceutical formulations.

However, a central question remains: How do *R. philippinarum* polysaccharides mediate their pharmacological activity *in vivo* following oral administration? Although oral intake is the most common route of administration for polysaccharide-based products, key aspects regarding their absorption, distribution, metabolism, excretion (ADME), and mechanistic pathways remain poorly understood, despite the well-documented physiological outcomes. It is well known that the pharmacokinetic behaviors of polysaccharide were closely correlated with their biological activities and physiological effects [14,15]. Furthermore, elucidating the pharmacokinetic profiles of polysaccharides can provide critical insights into how they exert their effects in the body [16].

To address this knowledge gap, direct *in vivo* tracing methodologies that enable real-time monitoring of polysaccharide fate are therefore essential. Currently, several methodologies have been employed to investigate the pharmacokinetics of marine-derived compounds, including fluorescence labeling [17,18], radioisotopic labeling, immunological assay methods, and physiologically based pharmacokinetic (PBPK) modeling [19,20]. A comprehensive review of pharmacokinetic studies on marine-derived active compounds has shown that analytical methods for these compounds in biomaterials include fluorescein labeling, radioactive labeling, HPLC or LC with various detection modes (fluorescence, UV, ELSD, or MS), and enzyme-linked immunosorbent assay (ELISA)-based approaches, with biomarker-based assays providing notably high sensitivity and broad linearity [21]. Immunological methods may suffer from detection errors due to metabolic fragmentation of antigenic determinants, limiting their sensitivity. Radioisotopic labeling offers high sensitivity but is restricted by short half-lives and radioactive hazards. PBPK modeling, while powerful for elucidating compartmental transfer kinetics and tissue-specific accumulation mechanisms of waterborne compounds in marine organisms [19,20], is less suited for directly quantifying the absorption and biodistribution of orally administered macromolecules such as polysaccharides. In contrast, fluorescent labeling offers enhanced sensitivity, operational simplicity, and safety advantages, supporting its selection for the present study [21,22].

In this study, we employed fluorescence labeling techniques to investigate the plasma concentration kinetics, biodistribution, and elimination pathways of orally administered *R. philippinarum* polysaccharides obtained by enzymatic hydrolysis (ERPP). A dual-labeling strategy was adopted: 5-DTAF, a visible fluorescent probe, was used for quantitative pharmacokinetic analysis in plasma and tissues, while the near-infrared dye Cy5.5 was employed for real-time whole-body NIR imaging given its superior tissue penetration and low autofluorescence background, enabling complementary quantitative and qualitative characterization of ERPP’s *in vivo* fate. The aims of this study were to: (1) develop and validate a fluorescence-based HPGPC-FLD method for quantitative tracking of ERPP in biological matrices; (2) characterize the plasma pharmacokinetics, tissue distribution, and excretion pathways of orally administered ERPP in rodent models; and (3) visualize real-time biodistribution using NIR imaging with a Cy5.5-labeled conjugate. Together, these investigations are intended to establish a pharmacokinetic framework for ERPP to support its further preclinical evaluation.

## 2. Results

### 2.1. Characterization of ERPP-F and ERPP-Cy5.5

**Figure 1:**
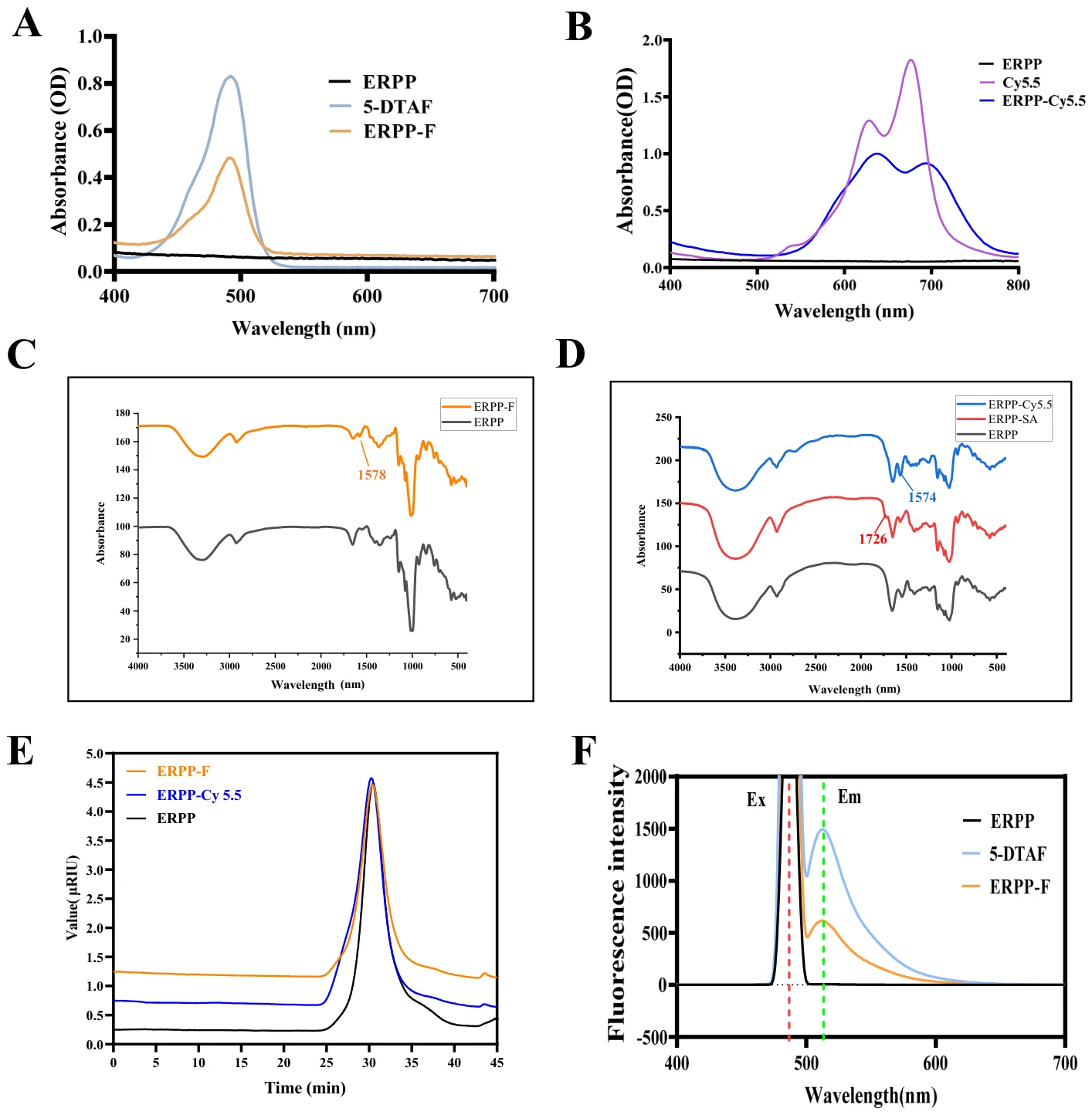
Characterization of fluorescently labeled ERPP. (**A**) The UV spectra of ERPP, 5-DTAF and ERPP-F. (**B**) The UV spectra of ERPP, Cy5.5 and ERPP-Cy5.5. (**C**) The FT-IR spectra of ERPP and ERPP-F. (**D**) The FT-IR spectra of ERPP, ERPP-SA and ERPP-Cy5.5. (**E**) The HPGPC-RID chromatograms of ERPP, ERPP-F and ERPP-Cy5.5. (**F**) The fluorescence spectrum of ERPP, 5-DTAF and ERPP-F, the red dotted line represents the 488 nm excitation light, and the green dotted line represents the 518 nm maximum emission spectral line.

ERPP-F and ERPP-Cy5.5 were characterized by ultraviolet spectrophotometer, fluorescence spectroscopy, infrared spectroscopy, and HPGPC-RID analysis. As shown in Figure 1A, a characteristic absorption peak was observed at 495 nm for 5-DTAF [23]. Similarly, ERPP-F also exhibited a characteristic absorption peak at 495 nm. Cy5.5 displayed characteristic absorption peaks at 630 and 680 nm [24]. Similarly, the ERPP-Cy5.5 conjugate showed two absorption peaks at identical wavelengths, confirming the successful labeling (Figure 1B). The slight differences between the absorption spectra of free Cy5.5 and ERPP-Cy5.5 are consistent with changes in the local microenvironment of the dye following covalent conjugation, a phenomenon commonly reported for dye–macromolecule conjugates. As illustrated in Figure 1C, the FT-IR spectrum of ERPP-F showed a characteristic absorption band at 1578 cm^−1^, which is typical of 5-DTAF. In Figure 1D, the peak at 1726 cm^−1^ was attributed to the carbonyl stretching vibration resulting from the conjugation of ERPP with SA. Meanwhile, the absorption at 1574 cm^−1^ corresponded to the C=C stretching vibration of the Cy5.5 dye. As depicted in Figure 1E, the molecular weight and distribution profiles of both ERPP-Cy5.5 and ERPP-F were found to be nearly identical to that of unmodified ERPP. Furthermore, 5-DTAF exhibited a characteristic emission peak near 516 nm [23].

As presented in Figure 1F, unlabeled ERPP produced no detectable emission, indicating the absence of inherent fluorescence. In contrast, the emission peak observed at approximately 517 nm for ERPP-F, which further confirmed the successful conjugation of 5-DTAF to ERPP. These findings confirmed the successful labeling of ERPP with both 5-DTAF and Cy5.5, thereby facilitating the subsequent experimental investigations. Successful removal of unbound dye following Sephadex G-25 purification was confirmed by multi-wavelength column elution profiling (Figure S2A,B). HPGPC-FLD analysis further confirmed that the fluorescent peaks of ERPP-F and ERPP-Cy5.5 co-eluted with the respective high-molecular-weight polysaccharide fractions and were clearly resolved from free 5-DTAF (~20 min) and free Cy5.5 (~35 min), respectively (Figure S2C,D), confirming the absence of unbound dye in the purified conjugates.

### 2.2. Determination of Fluorescence Substitution Degree of ERPP-F and ERPP-Cy5.5

The 5-DTAF and Cy5.5 standard curve was established as y = 3360.6x − 133.41 (*R*^2^ = 0.9977) and y = 355.02x (*R*^2^ = 0.9987). The fluorescence intensity of ERPP-F was determined and applied to this equation. The subsequent calculation revealed a fluorescence substitution degree of 0.61%, indicating that 100 μg of labeled ERPP-F contained 0.61 μg of 5-DTAF. These specific substitution ratios were chosen as they provide an optimal equilibrium. The fluorescence intensity of ERPP-Cy5.5 was measured, and the degree of fluorescence substitution was calculated to be 0.472%, indicating that 100 μg of ERPP-Cy5.5 contained 0.48 μg of Cy5.5. They are sufficiently low to preserve the native architecture of the polysaccharide from alterations induced by the fluorophore, without compromising the detectability required for subsequent analytical techniques [22,25].

### 2.3. Verification of Quantitative Analysis Method of ERPP-F

#### 2.3.1. Establishment of Linear Relationship

A standard curve was constructed by plotting the concentration of ERPP-F (*X*-axis) against the corresponding fluorescence intensity (*Y*-axis). Excellent linearity was observed over the concentration range of 0.25–100 μg/mL for all biological matrices, with a correlation coefficient (*R*^2^) greater than 0.99. The linear range, regression equation, and correlation coefficient are summarized in Table 1, confirming that all criteria for linearity in quantitative analysis were satisfied.

#### 2.3.2. Precision Assessment

The inter-day and intra-day precision for all QC biological samples was found to remain below 7.55%, while recovery rates ranged from 93.98 ± 4.59% to 110.88 ± 7.83% (Table 2). In conclusion, the method employed for ERPP-F in diverse biological matrices exhibited favorable precision and satisfactory recovery rates. Importantly, no discernible matrix effect was observed, which ensured the reliability of the *in vivo* concentration assessment of ERPP-F [26].

#### 2.3.3. In Vivo Integrity Verification of ERPP-F in Biological Matrices

**Figure 2:**
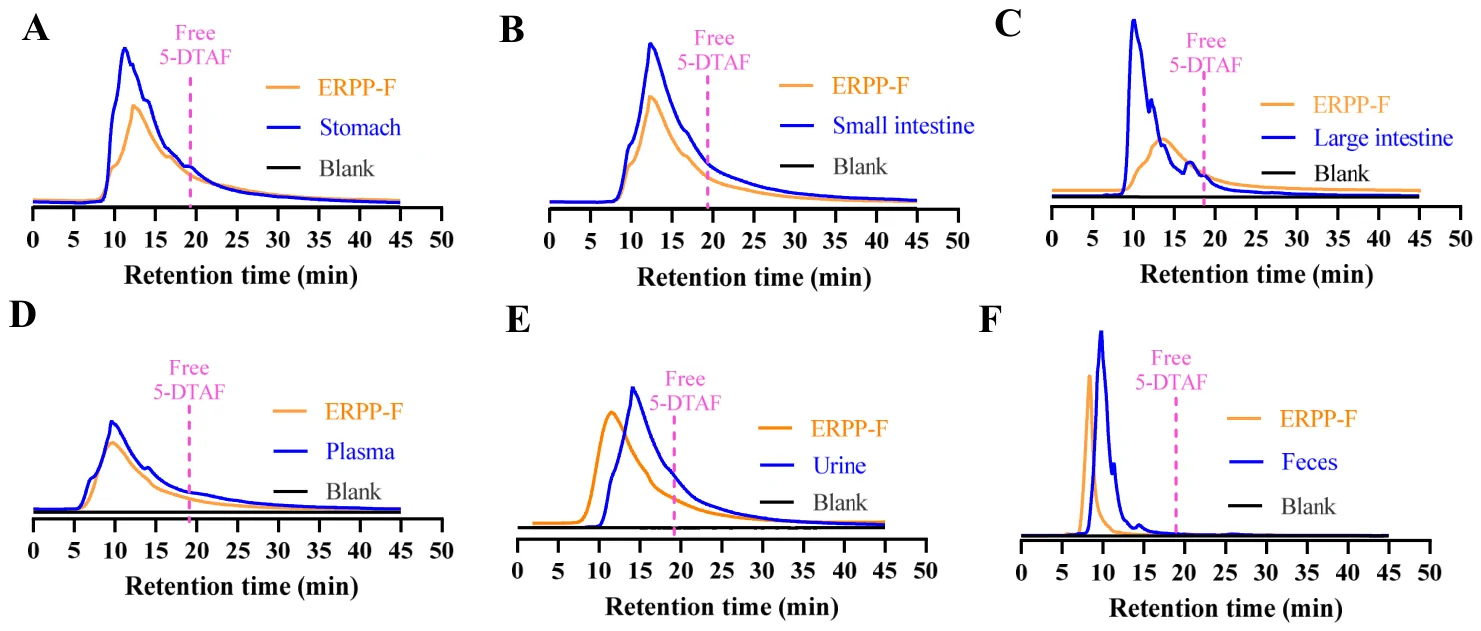
Representative chromatograms for the determination of ERPP-F in various bio-samples ((**A**): stomach; (**B**): small intestine; (**C**): large intestine; (**D**): plasma; (**E**): urine; (**F**): feces) by HPGPC-FLD. Black line: blank bio-samples; blue line: blank bio-samples spiked with standard ERPP-F solution; orange line: bio-samples collected after oral administration of ERPP-F; the dashed line indicates the retention time of free 5-DTAF.

The *in vivo* molecular status of ERPP-F was assessed by comparing HPGPC-FLD chromatograms of blank matrices, ERPP-F-spiked matrices, and biological samples collected after oral administration of ERPP-F (300 mg/kg) (Figure 2). In gastrointestinal contents and plasma, a single symmetrical peak was observed at approximately 10 min, consistent with intact high-molecular-weight ERPP-F. In fecal samples, the fluorescence peak co-eluted with the ERPP-F standard, indicating that the polysaccharide was predominantly excreted in an intact form. In contrast, the urinary peak shifted to a longer retention time, suggesting that only lower-molecular-weight fluorescent species derived from ERPP-F were detected in urine.

#### 2.3.4. Stability Assessment

**Figure 3:**
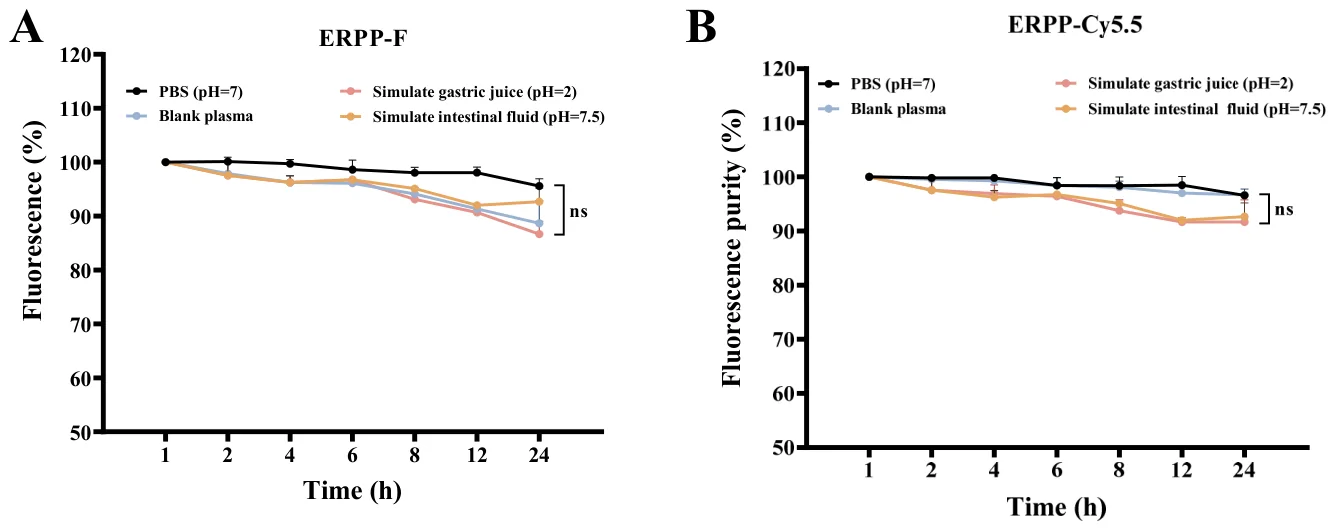
The stability of ERPP-F (**A**) and ERPP-Cy5.5 (**B**) in PBS, blank plasma, and the simulated gastric fluid, simulated intestinal fluid, PBS, and plasma by HPGPC-FLD. ns: No significant difference compared to the PBS (pH = 7). The stability of ERPP-F and ERPP-Cy5.5 under different conditions. Data are presented as mean ± SD (*n* = 3). Statistical significance was analyzed using one-way ANOVA followed by Tukey’s multiple comparison test.

The stability testing results are presented in Figure 3A. The fluorescence purity of ERPP-F was measured over a 24 h period in PBS, blank plasma, simulated gastric juice, and simulated intestinal fluid. The measured fluorescence purities were 95.6 ± 1.38%, 88.67 ± 4.51%, 86.67 ± 2.48%, and 92.67 ± 2.74%, respectively. Similarly, the fluorescence purity of ERPP-Cy5.5 under the same conditions was found to be 96.6 ± 0.36%, 96.17 ± 1.25%, 96.45 ± 1.45%, and 92.02 ± 4.04%, respectively (Figure 3B). The fluorescence purity was maintained above 90% in all cases, and this high stability was consistent with previous studies on polysaccharides [27]. The aforementioned experimental results demonstrated that the method exhibited satisfactory stability, precision, and accuracy, thereby fulfilling all requirements for pharmacokinetic studies.

### 2.4. Cytotoxicity Evaluation of ERPP, ERPP-F, and ERPP-Cy5.5 in Caco-2 and NCM460 Cells

**Figure 4:**
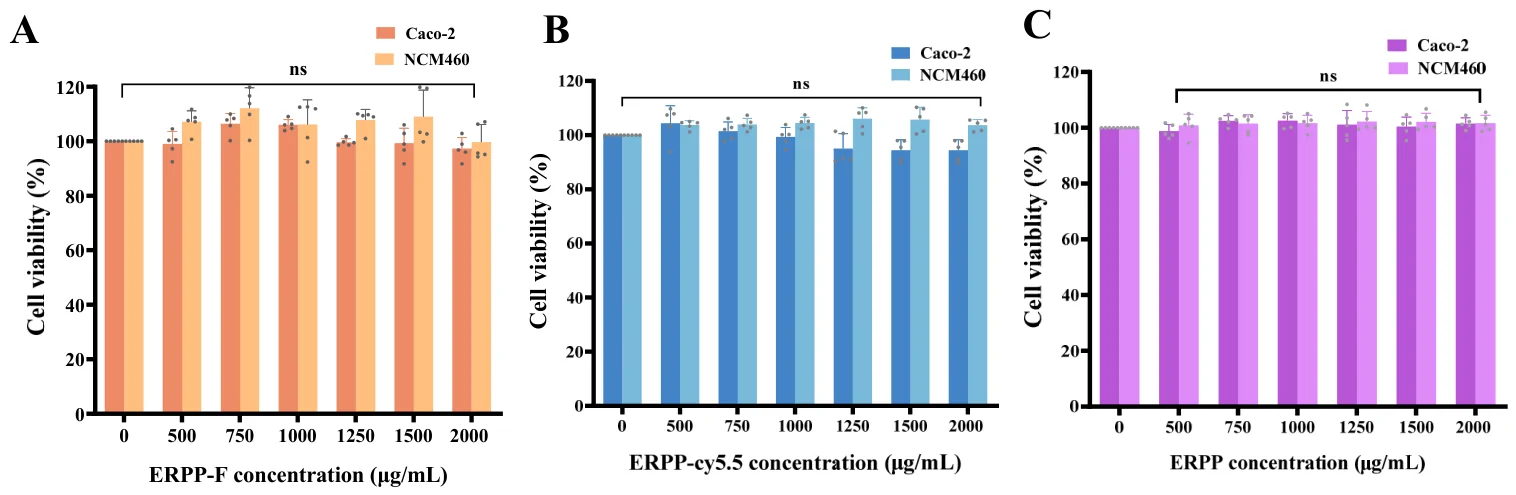
Cytotoxicity assays of Caco-2 and NCM460 cells treated with ERPP-F (**A**), ERPP-Cy5.5 (**B**), and ERPP (**C**). ns: No significant difference compared to the 0 μg/mL group. Data are presented as mean ± SD (*n* = 5). Statistical analysis was performed using one-way ANOVA followed by Tukey’s multiple comparison test.

The cytotoxicity of ERPP, ERPP-F (succinylated ERPP), and ERPP-Cy5.5 against Caco-2 and NCM460 cells after 48 h of treatment was evaluated using the CCK-8 assay (Figure 4). Cell viability remained above 95% at all tested concentrations for each preparation. According to the established criterion, samples with cell viability exceeding 80% are generally considered to have no significant cytotoxicity [28]. No significant differences in cell viability were observed among the three groups at any concentration tested (*p* > 0.05), suggesting that succinylation and Cy5.5 conjugation did not introduce measurable cytotoxic effects under the tested conditions. It should be noted, however, that cytotoxicity represents only one aspect of biological activity; the retention of other previously reported bioactivities of ERPP after chemical modification was not assessed in the present study. Dedicated functional assays are required to determine whether these biological activities are retained after chemical modification.

### 2.5. Real-Time Biodistribution of ERPP-Cy5.5 and Cy5.5 In Vivo

**Figure 5:**
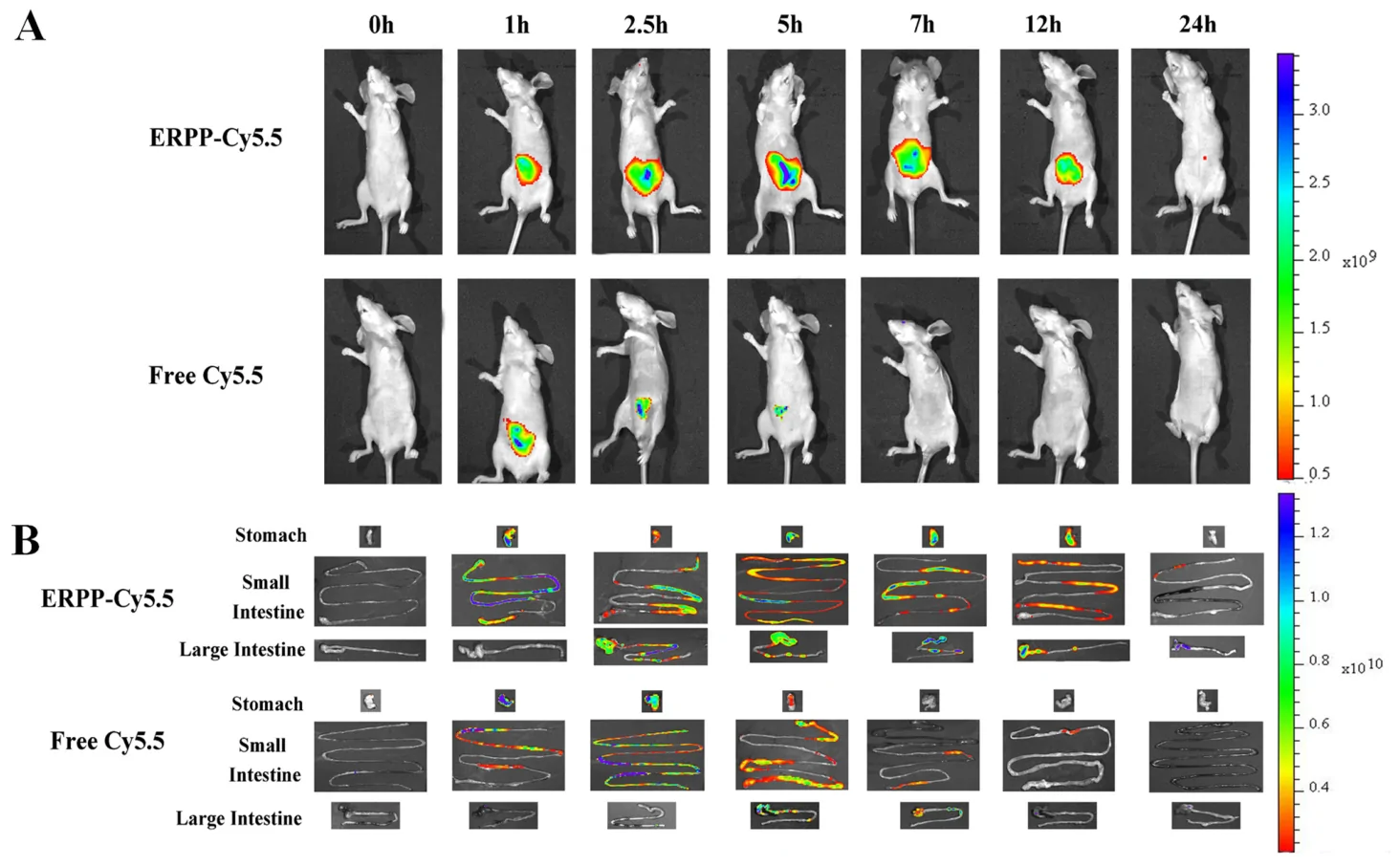
*In vivo* fluorescence imaging of ERPP-Cy5.5 and free Cy5.5 in mice following oral administration over a 24 h period. (**A**) *In vivo* imaging of the mice after oral administration of ERPP-Cy5.5 and free Cy5.5. (**B**) *Ex vivo* imaging of the excised intestine after oral administration of ERPP-Cy5.5 and free Cy5.5. Data are presented as mean ± SD (*n* = 3).

As shown in Figure 5A and Figure S4, following oral administration of ERPP-Cy5.5, fluorescence signals were predominantly concentrated in the abdominal region, peaking in intensity at 2.5–5 h and gradually diminishing thereafter, with residual signal still detectable at 24 h. In contrast, mice receiving free Cy5.5 at an equivalent molar dose exhibited a markedly weaker and more transient abdominal signal, which declined rapidly after 2.5 h and was largely undetectable by 7 h. This pronounced difference in signal intensity and duration between the two groups indicates that conjugation of Cy5.5 to ERPP substantially prolongs gastrointestinal retention and systemic exposure, consistent with the large molecular weight of the polysaccharide carrier.

*Ex vivo* gastrointestinal imaging further revealed distinct transit dynamics between the two groups (Figure 5B). In the ERPP-Cy5.5 group, fluorescence was detected throughout the stomach and small intestine within 1 h of administration, with the signal progressively migrating distally from the duodenum and jejunum toward the cecum and large intestine over the 2.5-7 h period, consistent with sustained GI transit of the intact polysaccharide. By 24 h, intestinal fluorescence had largely cleared. In the free Cy5.5 group, small intestinal fluorescence was weaker and more heterogeneous, appearing as discrete punctate signals rather than a continuous distribution, and diminished rapidly after 5 h with minimal signal in the large intestine. These differences suggest that free Cy5.5 is absorbed or cleared more rapidly from the GI tract, whereas ERPP-Cy5.5 undergoes slower, more prolonged intestinal transit characteristic of a high-molecular-weight polysaccharide.

*Ex vivo* organ imaging confirmed distinct biodistribution patterns between groups (Figure S3). In the ERPP-Cy5.5 group, strong hepatic and renal fluorescence was observed from 1 h and sustained through 24 h, with transient splenic and pulmonary signals peaking at 5 h. The free Cy5.5 control showed rapid hepatic signal decay after 5 h and minimal organ retention by 24 h, with no detectable splenic or pulmonary signal. Notably, brain fluorescence was detected in both groups with comparable intensity, and appeared earlier in the free Cy5.5 control (1 h vs. 2.5 h), indicating that the brain signal originates from CNS penetration of free Cy5.5 dye rather than intact ERPP-Cy5.5 crossing the blood–brain barrier. More broadly, it should be acknowledged that the ERPP-Cy5.5 group signals detected in the liver, kidneys, spleen, and lungs likely represent a composite of intact conjugate-associated fluorescence and a free Cy5.5 background contribution, given that free Cy5.5 was detectable in all organs examined in the control group. The ERPP-Cy5.5 group showed substantially higher and more sustained organ-level signals compared to the free Cy5.5 control (Figure S3), suggesting that the majority of the organ signal in the ERPP-Cy5.5 group reflects the polysaccharide conjugate; however, a free-dye contribution cannot be excluded.

### 2.6. Pharmacokinetic Parameters of ERPP-F in Rats

**Figure 6:**
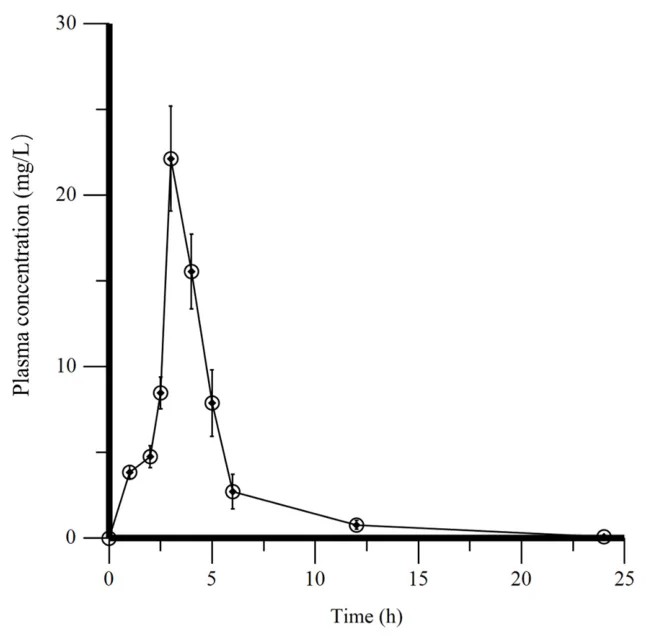
Plasma concentration–time profile of ERPP-F after oral administration (300 mg/kg) in rats (*n* = 6). Data are presented as mean ± SD.

As shown in Figure 6, ERPP-F was detectable in the plasma within 1 h after administration and reached its maximum concentration at 3 h, and then exhibited a gradual decline. The corresponding pharmacokinetic parameters were derived by fitting the concentration–time curve and a non-compartmental pharmacokinetic model. As summarized in Table 3, a C_max_ of 22.7 ± 2.7 mg/L was achieved within 3 h. The CL/F was determined to be 4.6 ± 0.3 L/h/kg, and the T_1/2_ was 3.2 ± 1.3 h. The MRT was 3.9 ± 0.5 h, and the AUC was 65.8 ± 3.9 h·mg/L. These results indicate that ERPP-F was detectable for up to 24 h following oral administration. These pharmacokinetic characteristics are consistent with those reported for pumpkin polysaccharides of comparable molecular weight [29].

### 2.7. Distribution of ERPP-F in Different Tissues

The 2.5 h time point was selected to capture the absorption phase (prior to T_max_), and 5 h to capture the post-absorptive distribution/elimination phase. ERPP-F was predominantly concentrated in the gastrointestinal tract at 2.5 h, suggesting two potential roles within the digestive system (Table 4). A portion of ERPP-F may be absorbed into the bloodstream, while another part may be utilized by the intestinal microbiota. Furthermore, ERPP-F was detectable in all examined tissues, with relatively higher levels observed in the liver, kidneys, and lungs compared to other biological samples. These findings were consistent with the qualitative NIR imaging observations in mice.

### 2.8. Excretion of ERPP-F

**Figure 7:**
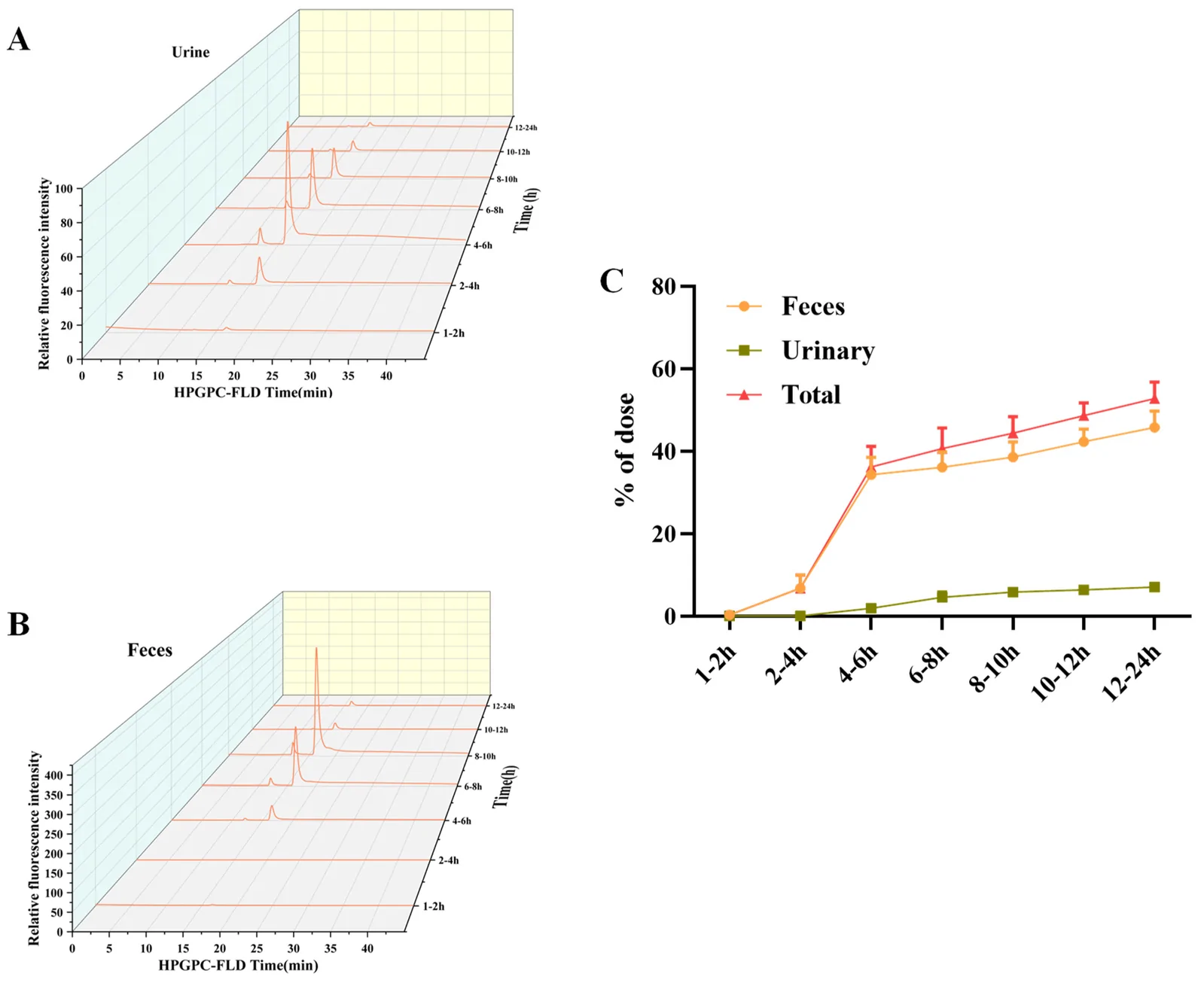
Excretion of ERPP-F in urine and feces after oral administration in rats (*n* = 5). (**A**) Changes in ERPP-F fluorescence intensity in urine over time. (**B**) Changes in ERPP-F fluorescence intensity in feces over time. (**C**) Cumulative excretion of ERPP-F after oral administration. Data are expressed as mean ± SD (*n* = 5). Statistical significance of urinary and fecal excretion data was determined using two-way ANOVA followed by Bonferroni’s multiple comparisons test. *p* < 0.05 was considered statistically significant.

Urinary excretion peaked within 4–6 h, declined rapidly thereafter, and was largely completed within 12 h (Figure 7A). In contrast, fecal excretion started after a 4–6 h lag, reached its maximum at 8–10 h, and then gradually decreased (Figure 7B). Within the 24 h observation window, fecal excretion accounted for the majority of recovered dose (45.7 ± 4.4%), substantially exceeding urinary excretion (7.0 ± 1.4%), suggesting that fecal elimination is likely the predominant route. However, as cumulative recovery curves for both urine and feces had not reached a plateau by 24 h (Figure 7C), the total excretion profile remains incomplete. Extended collection periods are required to confirm fecal excretion as the primary elimination pathway and to establish complete mass balance.

## 3. Discussion

The antioxidant, immunomodulatory, and anti-tumor activities of *R*. *philippinarum* polysaccharides are well-documented from our previous work [4,5,13]. A comprehensive understanding of their ADME properties, however, is still lacking. The conventional view holds that these polysaccharides are not easily absorbed and mostly degraded by intestinal microorganisms, which has limited the exploration of their systemic effects. However, accumulating evidence indicates that polysaccharides can be absorbed intact via multiple intestinal pathways, including endocytosis by M cells and intestinal epithelial cells, as well as paracellular transport [30]. To directly visualize these potential absorption routes and trace the subsequent *in vivo* processes, techniques such as fluorescent labeling, radioisotopic labeling [31], and immunoassay [32] have been developed. In this study, we utilized a fluorescence-based strategy by labeling ERPP with Cy5.5 and 5-DTAF, enabling real-time tracking of its *in vivo* fate.

The sensitivity and reproducibility of the HPGPC-FLD method established here are comparable to, or in some aspects exceed, those reported in previous pharmacokinetic studies of fluorescently labeled polysaccharides. The linear range of 0.25–100 μg/mL achieved in our study is broader than that reported for *Radix Ophiopogonis* polysaccharide using a similar HPGPC-FLD approach (0.25–20.0 or 50.0 μg/mL), and the intra- and inter-day precision (RSD < 7.55%) is superior to that reported for the same polysaccharide (RSD < 10%) [14]. In comparison with radioisotopic labeling methods, which typically achieve higher absolute sensitivity but require specialized facilities and raise safety concerns, the fluorescence labeling approach employed here offers comparable quantitative precision with greater operational accessibility and no radioactive hazard. Biomarker-based assays, as reviewed by Shikov et al. [21], can provide broad linearity (e.g., 0.014–1.13 mg/mL for fucoidan by anti-Xa activity assay) but are compound-specific and cannot be generalized to ERPP, which lacks an established biomarker. ELISA-based methods offer high specificity but require specific antibodies and may suffer from cross-reactivity with metabolic fragments. The HPGPC-FLD method therefore offers a practical balance of sensitivity, selectivity, and versatility for tracking intact polysaccharide species across multiple biological matrices simultaneously.

In pharmacokinetic studies, natural polysaccharides are frequently labeled through reductive amination reactions. However, the structural integrity and biological activity of polysaccharides may be compromised under the relatively stringent conditions required for these reactions [33]. Based on previous studies, two alternative labeling methods were adopted in this work rather than reductive amination. As displayed in Figure 1, NIR imaging was performed using ERPP labeled with the NIR dye Cy5.5. To facilitate conjugation, carboxyl groups were initially introduced into ERPP by a reaction with SA, yielding ERPP-SA. The carboxyl groups on ERPP-SA were then activated by EDC/NHS, enabling the formation of amide bonds with Cy5.5 and thereby successfully achieving conjugation. In a carbonate buffer (pH 9.5), a nucleophilic substitution reaction between the hydroxyl groups of ERPP and 5-DTAF resulted in covalent conjugation. The resulting conjugate exhibited sufficient chemical stability under the experimental conditions used in this study, as supported by the stability evaluation described below. Comprehensive characterization using UV-Vis spectroscopy, FT-IR, fluorescence spectroscopy, and HPGPC-FLD supported the successful incorporation of both Cy5.5 and 5-DTAF into ERPP. It should be noted that succinylation introduces anionic carboxyl groups onto ERPP prior to Cy5.5 conjugation, rendering ERPP-Cy5.5 a chemically modified derivative with potentially altered surface charge and hydrophilicity relative to native ERPP. Therefore, the NIR biodistribution data reflect the *in vivo* fate of this derivative, which may not fully represent unmodified ERPP.

By utilizing the established fluorescent probes, we conducted a visual exploration of these complex *in vivo* processes. Our pharmacokinetic investigation yielded quantitative insights into the dynamic behavior of ERPP. Following oral gavage, ERPP-F reached a peak plasma concentration (C_max_ = 22.7 ± 2.7 mg/L) at 3 h post-administration and remained quantifiable for up to 24 h, with a terminal half-life of 3.2 ± 1.3 h and MRT of 3.9 ± 0.5 h. Non-compartmental analysis estimated an AUC (0-t) of 65.8 ± 3.9 mg/L·h, indicating measurable systemic exposure following oral administration. The highest tissue concentrations were observed in the gastrointestinal tract, which is consistent with the oral absorption route. Recent evidence demonstrates that glucan-type polysaccharides can penetrate intestinal epithelial cells and reach the liver via clathrin heavy chain (CLTC) and dynamin1/Rab5-dependent endocytosis, providing a possible mechanistic explanation for the hepatic distribution observed in the present study [34]. It should be noted that the NIR imaging study in mice (5 mg/kg ERPP-Cy5.5) and the quantitative pharmacokinetic study in rats (300 mg/kg ERPP-F) were performed under different conditions (species, dose, and fluorescent label). Therefore, the temporal profiles of fluorescence signals cannot be directly compared or quantitatively correlated with the plasma concentration–time curve (e.g., T_max_ at 3 h). The two datasets serve complementary purposes: real-time spatial tracking versus precise kinetic quantification. This distribution pattern is consistent with the reports regarding polysaccharides from *Grifola frondosa* [35], *Smilax china* [36], and *Angelica sinensis* [37]. Strong hepatic and renal fluorescence were observed in the ERPP-Cy5.5 group from 1 h and sustained through 24 h, substantially exceeding the free Cy5.5 control signal. While a free-dye background contribution cannot be excluded in any organ, the markedly distinct biodistribution pattern compared with the free Cy5.5 control suggests that the organ-level signals in the ERPP-Cy5.5 group predominantly reflect the polysaccharide conjugate rather than free dye alone. Considering the distribution of ERPP in the lungs, spleen, and kidneys, potential research directions may involve exploring its immunomodulatory effects, splenocyte protection, and nephroprotective functions.

Fecal excretion was identified as the predominant elimination route, accounting for 45.7 ± 4.4% of the recovered dose within the 24 h observation window, representing the main pathway for intact or minimally degraded polysaccharide. The intact-ERPP claim rests primarily on this fecal fraction, which HPGPC-FLD confirmed to co-elute with the intact ERPP-F standard (Figure 2F). Urinary excretion (7.0 ± 1.4%) comprised exclusively low-molecular-weight fluorescent species, as intact ERPP (~4.65 × 10^5^ Da) exceeds the glomerular filtration threshold (~65 kDa) and cannot reach the urinary filtrate directly. However, it should be noted that mass balance had not reached a plateau by 24 h, indicating that the total excretion profile has not been fully characterized. Future studies should extend the collection period until cumulative recovery reaches a plateau, allowing complete mass balance to be established. It should be acknowledged that detailed glycosidic linkage and branching characteristics of ERPP were not determined in the present study and remain to be investigated in future work.

## 4. Materials and Methods

### 4.1. Materials

Cyanine 5.5 amine (Cy5.5) was purchased from Lumiprobe Corporation (Hallandale Beach, FL, USA). 5-([4,6-dichlorotriazin-2-yl] amino) fluorescein (5-DTAF) was purchased from Aladdin Chemical Co. (Shanghai, China). Trypsin was obtained from Deebio Pharmaceutical Co., Ltd., (Sichuan, China). Succinic anhydride (SA), Dimethylaminopyridine (DMAP), N-hydroxysuccinimide (NHS), and 1-(3-dimethylaminopropyl)-3-ethyl carbodiimide hydrochloride (EDC) were provided by Aladdin Chemical Co. (Shanghai, China). CCK-8 kit was purchased from Beyotime Biotechnology (Shanghai, China). All other chemical reagents used in the study were of analytical grade.

### 4.2. Preparation of ERPP

ERPP was obtained and structurally characterized as described in our previous study [4]. Briefly, the *Ruditapes philippinarum* samples were purchased from a local seafood market in Qingdao, China. The species was zoologically identified by Professor Zhao-Ping Wang (College of Fisheries, Ocean University of China). A voucher specimen (QDU-2025-ERPP) has been deposited in the Herbarium Room of the College of Life Sciences, Qingdao University. The meat of *R. philippinarum* was mixed with water at a 1:2 ratio and homogenized. Subsequently, trypsin was added to facilitate enzymatic hydrolysis for 3 h, after which the mixture was centrifuged. The supernatant was further processed using a 30 kDa ultrafiltration membrane. Precipitation was achieved by adding ethanol to the concentrated solution. Finally, the precipitate was separated by chromatography using DEAE-52 and HW-65 to obtain ERPP with a weight-average molecular weight of 4.65 × 10^5^ Da.

Monosaccharide composition was determined by PMP-HPLC following acid hydrolysis. Briefly, monosaccharide standards, including rhamnose, arabinose, galactose, glucose, xylose, mannose, glucuronic acid, galacturonic acid, glucosamine hydrochloride, galactosamine hydrochloride, and fucose, were prepared for calibration at concentrations of 10–500 µg/mL, except for fucose, which was prepared at 20–1000 µg/mL. ERPP (5 mg) was hydrolyzed with 2 M TFA at 110 °C for 6 h. After evaporation under nitrogen, the hydrolysate was derivatized with 1-phenyl-3-methyl-5-pyrazolone (PMP) in NaOH at 70 °C for 1 h, extracted with chloroform, and the aqueous phase was analyzed by HPLC (Thermo U3000, Thermo Fisher Scientific, Waltham, MA, USA) on a ZORBAX Eclipse XDB-C18 column (4.6 × 250 mm, 5 μm; Agilent Technologies Inc., Santa Clara, CA, USA) at 30 °C with a mobile phase of 0.05 M phosphate buffer/acetonitrile (83:17, *v*/*v*) at 0.8 mL/min, detected at 250 nm. Monosaccharide composition analysis by PMP-HPLC revealed that ERPP is composed predominantly of glucose (97.95%), with trace amounts of galactose (0.65%), mannose (0.63%), glucosamine (0.73%), and galactosamine (0.04%) (Figure S1, Table S1).

### 4.3. Preparation of ERPP-Cy5.5 and ERPP-F

**Figure 8:**
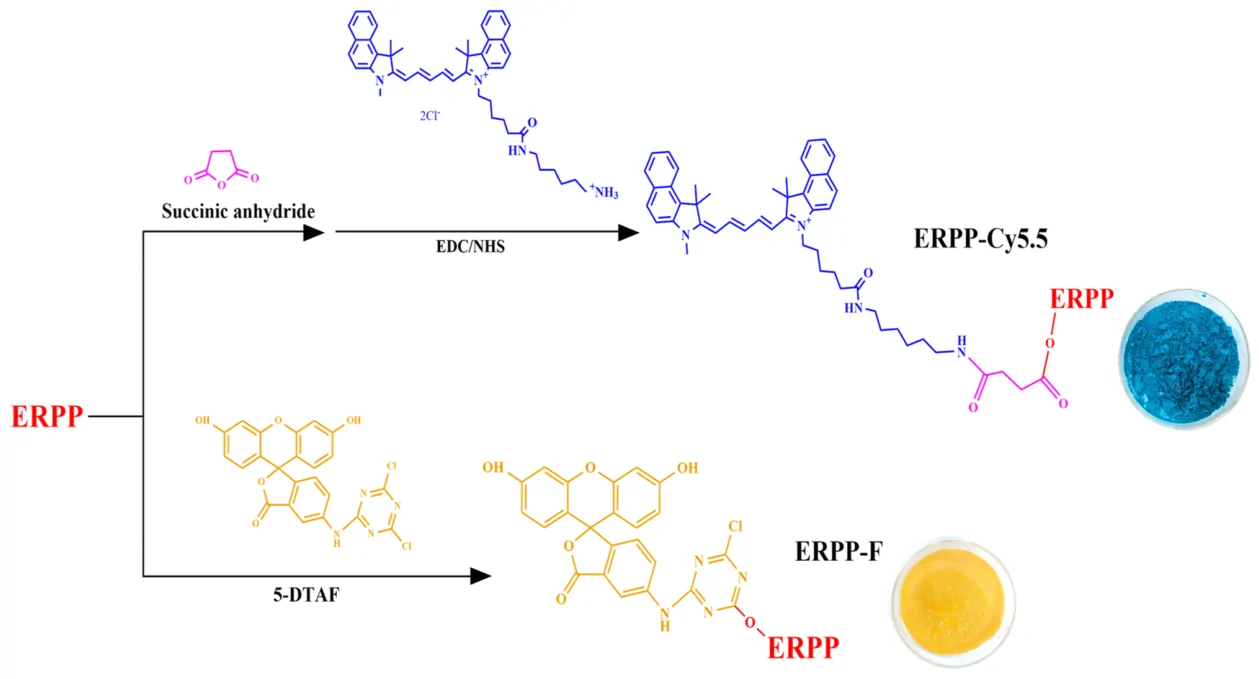
The schematic diagram of labeling reaction.

As shown in Figure 8, the labeling method of Cy5.5 was referred to previous studies [29]. Briefly, ERPP (800 mg) was dissolved in ultrapure water and reacted with SA (800 mg) and DMAP (800 mg) at 45 °C for 22 h to introduce carboxyl groups, yielding ERPP-SA after dialysis and lyophilization. ERPP-SA (500 mg) was then activated with EDC (600 mg) and NHS (500 mg) in PBS at 0 °C for 2 h, followed by slow addition of Cy5.5 amine (24 mg, dissolved in 1 mL DMF) under alkaline conditions (pH 8.5). The reaction proceeded at room temperature for 48 h in the dark, after which the product was dialyzed, lyophilized, and further purified by Sephadex G-25 chromatography to obtain ERPP-Cy5.5.

Fluorescent labeling of ERPP with 5-DTAF was carried out by adapting a published method [25]. Briefly, ERPP (500 mg) was dissolved in carbonate buffer (200 mL, pH 9.5) and reacted with 5-DTAF (40 mg, dissolved in 15 mL carbonate buffer) by alternating stirring at 25 °C and 4 °C for 24 h. Unbound dye was removed by dialysis against distilled water, and the product was further purified by Sephadex G-25 chromatography and lyophilized to yield ERPP-F.

Successful conjugation of fluorescent dyes to ERPP was demonstrated by UV-Vis spectroscopy (characteristic absorbance peaks at 495 nm for 5-DTAF and 630/680 nm for Cy5.5), FT-IR spectroscopy (new absorption bands at 1578 cm^−1^ in ERPP-F, and 1726/1574 cm^−1^ in ERPP-Cy5.5), fluorescence spectroscopy (retention of 5-DTAF excitation/emission characteristics), and HPGPC-FLD (co-elution of the fluorescent peak with the high-molecular-weight polysaccharide fraction, resolved from free dye). It should be noted that succinylation of ERPP with SA introduces additional anionic carboxyl groups into the polysaccharide backbone prior to Cy5.5 conjugation, which may alter the surface charge, hydrophilicity, and potentially the receptor-mediated recognition of ERPP-Cy5.5 relative to unmodified ERPP. Consequently, the biodistribution data obtained from NIR imaging reflect the *in vivo* fate of this succinylated derivative rather than native ERPP per se, and should be interpreted with this distinction in mind.

### 4.4. Characterization of ERPP, ERPP-F and ERPP-Cy5.5

Stock solutions of ERPP, ERPP-F, and ERPP-Cy5.5 (100 μg/mL) were prepared in PBS after accurate weighing. A Tecan Spark microplate reader (Tecan, Männedorf, Switzerland) was used to record UV-Vis spectra from 400 to 800 nm. Fluorescence spectral properties (excitation/emission) were analyzed with a Hitachi F-700 spectrophotometer (Hitachi High-Tech Corporation, Tokyo, Japan), scanning from 400 to 700 nm for 5-DTAF and ERPP-F. FT-IR spectra (4000–400 cm^−1^) were collected using a Thermo Nicolet 6700 spectrometer (Thermo Fisher Scientific, Madison, WI, USA).

ERPP-F and ERPP-Cy5.5 were purified by gel filtration chromatography with a Sephadex G-25 column (1.6 × 80 cm). Elution was performed with distilled water at a flow rate of 0.5 mL/min, with fraction collection set at 4.5 mL/tube. Polysaccharide-containing fractions were identified by the phenol–sulfuric acid assay (490 nm), while 5-DTAF and Cy5.5 conjugates were monitored by UV detection at their characteristic absorption wavelengths (495 nm and 680 nm, respectively).

### 4.5. Determination of Cytotoxicity of the ERPP-F and ERPP-Cy5.5

Caco-2 and NCM460 were obtained from the Cell Bank of the Chinese Academy of Sciences (Shanghai, China). Cells were cultured in DMEM supplemented with 10% fetal bovine serum and 1% penicillin–streptomycin at 37 °C in a humidified atmosphere containing 5% CO_2_. The cytotoxicity profiles of ERPP, ERPP-F and ERPP-Cy5.5 against Caco-2 and NCM460 cell lines were assessed through the CCK-8 assay. Cells were seeded in 96-well plates (4 × 10^4^ cells/well; 100 μL medium) and incubated for 12 h. The cells were then exposed to compound gradients (0, 500, 750, 1000, 1250, 1500, 2000 μg/mL), proceeding for 48 h at 37 °C. Post-treatment cell viability was quantified using the CCK-8 reagent according to manufacturer protocols.

### 4.6. Determination of the Degree of Fluorescence Substitution

Stock solutions of 5-DTAF and Cy5.5 (1 μg/mL in PBS) were serially diluted to concentrations ranging from 0.2 to 1 μg/mL, with equivalent PBS volumes serving as blank controls. Fluorescence intensities were measured using a spectrophotometer at the characteristic wavelengths: 5-DTAF (Ex = 490 nm/Em = 518 nm) and Cy5.5 (Ex = 680 nm/Em = 710 nm). Standard calibration curves were subsequently generated from these measurements, with regression equations derived for quantitative fluorescence analysis. Weighed preparations of ERPP-F and ERPP-Cy5.5 (1 mg in 10 mL PBS) were analyzed by fluorescence spectrophotometry using 1.8 mL aliquots, enabling quantitative determination of polysaccharide labeling efficiency to evaluate conjugation efficacy.

### 4.7. Establishment of Quantitative Analysis Method

Standard calibration curves were established by adding ERPP solutions (graded concentrations in PBS) into blank plasma, tissue homogenates, and fecal matrices. The calibration curve exhibited a linear range of 0.25–100 μg/mL. For method validation, quality control (QC) samples at low, medium, and high concentrations (0.5, 10, 25 μg/mL, *n* = 5) were used to assess precision and accuracy.

To assess the stability of the ERPP-5-DTAF conjugate under simulated physiological conditions, the stability of ERPP-F was evaluated in simulated gastric fluid (pH = 1.2), simulated intestinal fluid (pH = 7.4), PBS (pH = 7), and plasma at 37 °C over 24 h. The *in vitro* stability of ERPP-F (25 μg/mL) was evaluated under specific conditions. The sample was incubated at 37 °C in either 0.5 mL of phosphate-buffered saline (PBS) or plasma. Simulated gastric fluid (SGF) and small intestinal fluid were prepared according to established methods [29]. Specifically, the SGF was prepared by dissolving NaCl (3.1 g/L), KCl (1.1 g/L), NaHCO_3_ (0.6 g/L), and CaCl_2_ (0.15 g/L) in water, adjusting the basal medium to pH 2.5 with 0.1 M HCl, and then blending 150 g of this solution with 1 mL of CH_3_COONa (1.0 M, pH = 5.0) and 35.0 mg of pepsin to achieve a final pH = 2.0. Concurrently, the simulated small intestinal fluid was formulated using a basal medium containing NaCl (5.4 g/L), KCl (0.65 g/L), and CaCl_2_·2H_2_O (0.33 g/L) adjusted to pH = 7.0 with 0.1 M NaOH, which was subsequently mixed with 13 mg trypsin, 100 g pancreatin solution (7%, *w*/*w*), and 200 g bile salt solution (4%, *w*/*w*) to yield a final fluid adjusted to pH = 7.5. Subsequently, the stability evaluation in the simulated gastrointestinal media employed fluorescence intensity quantification at baseline (0 h) and time points of 1, 2, 4, 6, 8, 12 and 24 h. It should be noted that the simulated gastrointestinal fluids used in this study were prepared to evaluate the chemical stability of ERPP-F under gastric acid and pancreatic protease conditions, and did not include glycosidases. As a result, the stability data reported here do not capture potential glycosidase-mediated hydrolysis of the polysaccharide backbone that may occur *in vivo* through intestinal microbiota activity. This represents a limitation of the current stability assessment, and the contribution of microbial glycosidases to ERPP metabolism *in vivo* warrants further investigation.

### 4.8. In Vivo Pharmacokinetic Study

All animal experiments received approval from the Animal Care and Use Committee of Qingdao University (QDU-AEC-2025036) and were conducted in strict accordance with the NIH Guide for the Care and Use of Laboratory Animals. BALB/c mice (19 ± 2.4 g, 4–5 weeks old) and Wistar rats (200 ± 20 g, 6–8 weeks old) were procured from Vital River Laboratory Animal Technology (Beijing, China). The animals were housed in standard polypropylene cages under controlled environmental conditions (22 ± 2 °C, 12 h light/dark cycle) with ad libitum access to food and water. Following a one-week acclimatization period, subjects underwent a 12 h fast prior to experimental procedures.

#### 4.8.1. Plasma Pharmacokinetic Study

To comprehensively characterize its pharmacokinetic profile, a relatively high oral dose of 300 mg/kg was selected [29]. After dosing, serial blood samples (300 μL) were collected from the orbital plexus under brief isoflurane anesthesia at predetermined intervals (0, 1, 2, 3, 4, 6, 12, and 24 h). Samples were immediately transferred to heparinized tubes, centrifuged at 1000× *g* for 10 min (4 °C), and the resulting plasma was cryopreserved at −80 °C pending bioanalysis. Male Wistar rats (*n* = 6) were used for the plasma pharmacokinetic study. Animals were randomly assigned before experimentation. Blinding was applied during bioanalysis and data analysis.

#### 4.8.2. Tissue Distribution Study

Rats received a single oral dose of ERPP-F (300 mg/kg). At the designated time points (2.5 and 5 h), the rats were sacrificed, and specified organs, namely the liver, kidney, heart, lung, spleen, stomach, small intestine, large intestine, muscle, and thymus, were excised. Fluorescence intensity within each tissue sample was quantified to determine the biodistribution profile of ERPP-F. Male Wistar rats (*n* = 6) were used for the tissue distribution study. Animals were randomly assigned before experimentation. Blinding was applied during fluorescence measurement and data analysis.

#### 4.8.3. Real-Time Distribution of ERPP-Cy5.5 by NIR Imaging

NIR imaging was performed in female BALB/c mice following oral gavage administration of ERPP-Cy5.5 (5 mg/kg, dissolved in 0.3 mL ultrapure water). After a 12 h fasting period with ad libitum water access, real-time *in vivo* imaging was conducted at 0, 1, 2.5, 5, 7, 12, and 24 h post-administration with the IVIS Lumina XRMS III imaging system (PerkinElmer, Waltham, MA, USA; Ex: 680 nm, Em: 710 nm). Groups of mice (*n* = 3) were imaged sequentially under appropriate restraint at predetermined intervals. As a control, a separate group of mice (*n* = 3) received free Cy5.5 at a molar dose equivalent to that present in the ERPP-Cy5.5 conjugate (23.6 μg/kg) oral gavage under identical conditions and time points. The fluorescence intensity was quantified through region-of-interest (ROI) analysis using the Living Image® 4.2 software (PerkinElmer Inc., Waltham, MA, USA).

A high dose of 300 mg/kg was used in rats for quantitative pharmacokinetic studies to ensure detectable concentrations given the low oral bioavailability of polysaccharides. A low dose of 5 mg/kg was used in mice for NIR imaging to avoid fluorescence artifacts and non-specific retention while maintaining sufficient signal. These doses are not intended for direct comparison; the two datasets are complementary.

#### 4.8.4. In Vivo Fluorescence Integrity Verification

An SPD-20A fluorescence detector (Shimadzu Corporation, Kyoto, Japan) coupled to a gel permeation chromatography system (HPGPC-FLD) was employed to evaluate the *in vivo* integrity of ERPP-F across biological matrices. Analyte separation was achieved on a Shodex SB 805 HQ column (7.8 mm × 30.0 cm; Showa Denko K.K., Tokyo, Japan) at 30 °C using 0.1 M sodium nitrate as the isocratic mobile phase (0.5 mL/min). The fluorescence detector was operated at Ex/Em = 490/525 nm.

Biological samples including gastrointestinal contents (stomach, small intestine, large intestine), plasma, urine, and feces were collected at peak time points following oral administration of ERPP-F (300 mg/kg). For each matrix, chromatograms were obtained from blank biological matrix, blank matrix spiked with purified ERPP-F standard, and biological sample collected after oral dosing. Retention times were compared to determine whether the detected fluorescence corresponded to intact ERPP-F or low-molecular-weight species.

#### 4.8.5. Excretion Study

Wistar rats underwent a 24 h acclimatization period in individual metabolic cages prior to experimentation to enable quantitative urine and fecal collection. Following oral administration of ERPP-F (300 mg/kg), excretory samples were systematically collected at predetermined intervals (1-2, 2-4, 4-6, 6-8, 8-10, 10-12, and 12-24 h post-administration). Fluorescence intensity in urine and feces was measured to evaluate excretion kinetics. Male Wistar rats (*n* = 5) were used for the excretion study. Animals were randomly assigned before experimentation. Blinding was applied during fluorescence measurement and data analysis.

### 4.9. Pharmacokinetic and Data Analysis

The pharmacokinetic parameters of ERPP-F were obtained from the plasma concentration–time profiles and analyzed using a noncompartmental approach in Phoenix WinNonlin^®^ software (version 8.0; Certara USA, Inc., Princeton, NJ, USA). The comprehensive pharmacokinetic assessment included the following parameters: the maximum plasma concentration (C_max_), the time to C_max_ (T_max_), the elimination half-life (t_1/2_), the area under the concentration–time curve (AUC_0-t_), the area under the first moment curve (AUMC), the apparent systemic clearance (C_L/F_), the apparent volume of distribution (V_d/F_), and the mean residence time (MRT).

All data are expressed as mean ± standard deviation (SD). Data were analyzed and plotted with SPSS Statistics 21 (IBM Corp., Armonk, NY, USA) and GraphPad Prism 8.2.0 (GraphPad Software, Boston, MA, USA). For pharmacokinetic analysis, plasma concentration–time profiles were descriptively analyzed, and pharmacokinetic parameters, including C_max_, T_max_, AUC, and MRT, were calculated. For the multi organ tissue distribution and cumulative excretion data, statistical analysis was performed using a two-way ANOVA followed by Bonferroni’s multiple comparisons test. A *p*-value < 0.05 was considered statistically significant.

## 5. Conclusions

This study employed a dual-labeling strategy to investigate the pharmacokinetics and biodistribution of ERPP. The polysaccharide was first tagged with 5-DTAF via a mild nucleophilic substitution, followed by conjugation with the NIR dye Cy5.5 using SA chemistry. Successful labeling was verified by the characteristic spectroscopic signatures in UV, IR, and fluorescence analyses. Following oral gavage, ERPP-F achieved a peak plasma C_max_ of 22.7 ± 2.7 mg/L at 3 h (T_max_ = 3 h), with a half-life of 3.2 ± 1.3 h. Subsequent NIR imaging demonstrated the distribution pattern of the ERPP-Cy5.5 conjugate in mice, with the highest accumulation observed in the kidneys, liver, and lungs. Fecal excretion accounted for the largest recovered fraction within the 24 h observation window (45.7 ± 4.4% of the recovered dose), although longer collection periods are required to determine the complete elimination profile of ERPP. These findings establish a pharmacokinetic framework for ERPP and provide a pharmacokinetic basis to support its further evaluation as an oral therapeutic candidate.

## Figures and Tables

**Table 1:** Calibration curves for ERPP-F in rat plasma and tissues (*n* = 6).

Bio-Samples	Standard Curves	Correlation Coefficients	Linear Ranges (μg/mL)
Plasma	y = 94.227x + 299.98	0.9986	0.25–100
Heart	y = 10.576x + 40.384	0.9934	0.25–100
Liver	y = 16.918x + 2.509	0.9934	0.25–100
Spleen	y = 11.552x + 78.858	0.9966	0.25–100
Lung	y = 11.321x + 9.836	0.9988	0.25–100
Kidneys	y = 16.784x + 10.079	0.9951	0.25–100
Stomach	y = 11.727x + 69.054	0.9962	0.25–100
Small intestine	y = 9.8338x + 30.797	0.9954	0.25–100
Large intestine	y = 24.365x + 193.17	0.9919	0.25–100
Muscle	y = 12.183x + 14.591	0.9922	0.25–100
Thymus	y = 9.1059x + 58.577	0.9965	0.25–100
Feces	y = 38.563x + 202.94	0.9903	0.25–100
Urine	y = 4.5801x + 198.54	0.9996	0.25–100

**Table 2:** Precision and accuracy for the assay of ERPP-F in plasma and tissues (*n* = 5).

Bio-Samples	Concentration (μg/mL)	Accuracy (Mean ± S.D., %)	Precision (R.S. D., %)
Plasma	0.50	97.56 ± 2.29	2.35
	10.00	98.02 ± 1.61	1.64
	25.00	103.23 ± 4.96	4.80
Heart	0.50	96.24 ± 4.41	4.58
	10.00	97.66 ± 1.22	1.25
	25.00	99.75 ± 0.66	0.67
Liver	0.50	94.46 ± 6.66	7.05
	10.00	100.60 ± 1.04	1.03
	25.00	99.81 ± 0.54	0.54
Spleen	0.50	109.15 ± 6.18	5.66
	10.00	101.60 ± 3.66	3.61
	25.00	97.38 ± 6.80	6.98
Lung	0.50	93.98 ± 4.59	4.89
	10.00	98.24 ± 3.42	3.49
	25.00	103.88 ± 1.61	1.55
Kidneys	0.50	101.40 ± 7.65	7.55
	10.00	101.33 ± 0.58	0.57
	25.00	100.54 ± 0.70	0.69
Stomach	0.50	106.53 ± 1.30	1.22
	10.00	95.48 ± 0.61	0.64
	25.00	102.68 ± 1.39	1.35
Small intestine	0.50	103.27 ± 2.02	1.95
	10.00	105.81 ± 1.10	1.04
	25.00	103.71 ± 2.09	2.02
Large intestine	0.50	109.93 ± 4.98	4.53
	10.00	96.70 ± 3.13	3.24
	25.00	101.49 ± 2.41	2.38
Muscle	0.50	105.94 ± 2.79	2.63
	10.00	99.09 ± 0.77	0.78
	25.00	101.56 ± 1.52	1.49
Thymus	0.50	104.72 ± 2.93	2.80
	10.00	96.11 ± 2.26	2.35
	25.00	99.59 ± 2.36	2.37
Feces	0.50	94.63 ± 2.99	3.16
	10.00	99.95 ± 1.92	1.92
	25.00	105.08 ± 6.22	5.92
Urine	0.50	110.88 ± 7.88	7.10
	10.00	96.86 ± 1.42	1.47
	25.00	104.19 ± 3.27	3.14

**Table 3:** Pharmacokinetic parameters of ERPP-F after oral administration in rats (*n* = 6).

Pharmacokinetic Parameters	Units	i.g. (300 mg/kg)
AUC _(0-t)_	h*mg/L	65.8 ± 3.9
AUC _(0-ꝏ)_	h*mg/L	66.2 ± 3.7
AUMC _(0-t)_	h*h*mg/L	245.2 ± 23.0
AUMC _(0-ꝏ)_	h*h*mg/L	257.7 ± 30.4
MRT _(0-t)_	h	3.7 ± 0.3
MRT _(0-ꝏ)_	h	3.9 ± 0.5
V_d/F_	L/kg	21.5 ± 9.8
C_L/F_	L/h/kg	4.6 ± 0.3
T_max_	h	3.0
C_max_	mg/L	22.7 ± 2.7
Ke	1/h	0.3 ± 0.1
T_1/2_	h	3.2 ± 1.3

**Table 4:** *In vivo* biodistribution studies of ERPP-F in rats at 2.5 and 5 h (*n*=6).

Bio-Samples	2.5 h (μg/g Tissue)	5 h (μg/g Tissue)
Heart	6.60 ± 0.10	9.26 ± 0.08
Liver	20.40 ± 1.30	35.48 ± 0.02
Spleen	0.87 ± 0.04	1.00 ± 0.30
Lung	49.20 ± 0.60	20.00 ± 1.00
Kidneys	26.13 ± 0.03	39.05 ± 0.04
Stomach	585.00 ± 2.00	32.40 ± 0.50
Small intestine	7264.00 ± 36.00	942.00 ± 5.00
Large intestine	7.80 ± 1.20	352.20 ± 38.30
Muscle	<2.5	<2.5
Thymus	<2.5	<2.5

Values below the limit of quantification (LOQ) are presented as <2.5 µg/g tissue.

## Data Availability

The original contributions presented in the study are included in the article and Supplementary Materials; further inquiries can be directed to the corresponding author.

## Supplementary Materials

The following supporting information can be downloaded at https://www.mdpi.com/article/10.3390/md24080278/s1, Figure S1: The monosaccharide composition of ERPP. Table S1: Percentage of each component (%). Figure S2: Chromatographic and spectroscopic evidence for covalent conjugation and purification of fluorescently labeled ERPP. Figure S3: *Ex vivo* imaging of main organs after oral administration of ERPP-Cy5.5 and Cy5.5. Figure S4: The ROI value of abdomen, different gastrointestinal, and main tissues, after oral administration of ERPP-Cy5.5.
