# Supplementary material for: Pharmacokinetic Profiling Evaluation of Ruditapes philippinarum Polysaccharide
Source: Mar Drugs. 2026 Aug 11;24(8):278. doi: 10.3390/md24080278 (PMC13514109; doi:10.3390/md24080278)
Supplement: Supplementary file 1 [file marinedrugs-24-00278-s001.zip › marinedrugs-4441153-supplementary.pdf]

Supplementary material

Pharmacokinetic profiling evaluation of *Ruditapes philip-pinarum* polysaccharide

Meng-Yue Liu a, Dong-Li Yin b, Jia Yu a, Wei-Xia Wang b, Sheng-Can Zou b, Shuang Zhao a, Chun-Ze Zou c, Fei Li a * and Yu-Xi Wei a *

a College of Life Sciences, Qingdao University, Qingdao 266071, China

b Qingdao Yihai Industry Holdings Co., Ltd., Qingdao 266105, China

c Donald Bren School of Information and Computer Sciences, University of California, Irvine 92697, USA

Monosaccharide standards

Standard name	Retention time	Slope	R^2
1.Man	16.665	71.77	0.999
2.GlcN	20.842	87.66	0.999
3.Rha	23.387	33.61	0.995
4.GlcUA	24.447	66.96	0.999
5.GalUA	27.702	90.04	0.999
6.GalN	31.963	89.66	0.999
7.Glc	34.317	54.79	0.998
8.Gal	38.965	56.75	0.998
9.Xyl	41.617	48.27	0.995
10.Ara	43.082	71.59	0.994
11.Fuc	50.692	28.45	0.992

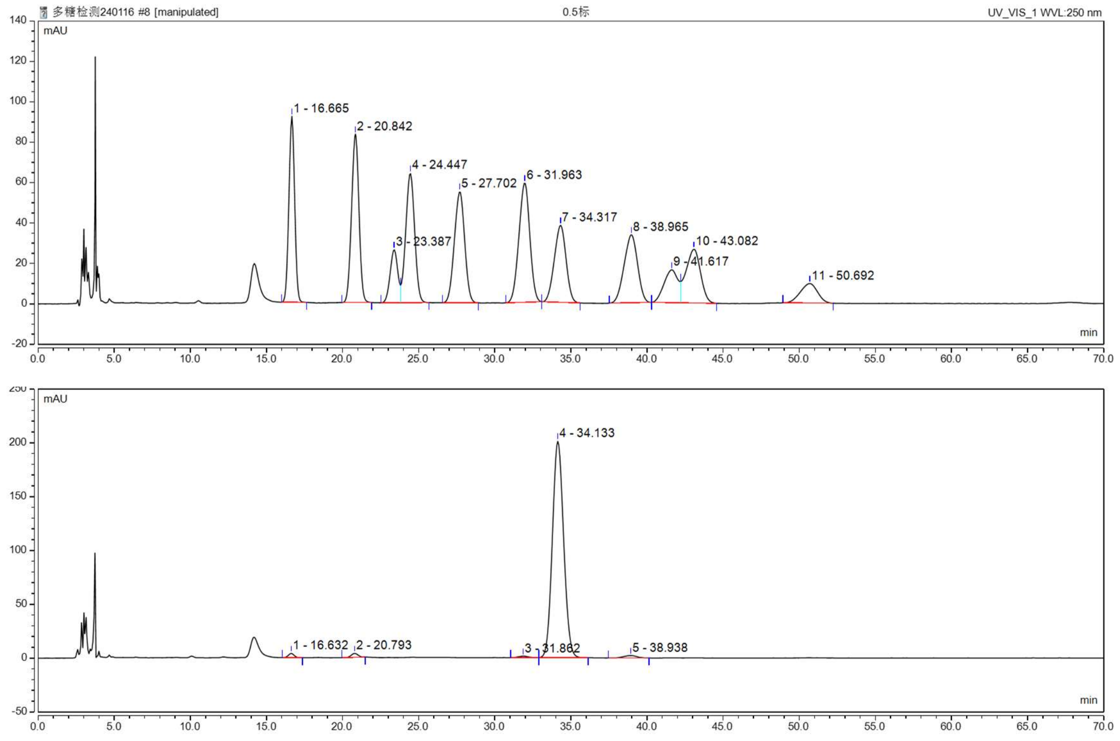

Figure S1: The monosaccharide composition of ERPP. (A): Standard sugar liquid chromatogram. (B): ERPP liquid chromatogram.

Table S1： Percentage of each component (%)

Percentage of each component (%)										
**Man**	**GlcN**	**Rha**	**GlcA**	**GalA**	**GalN**	**Glc**	**Gal**	**Xyl**	**Ara**	**Fuc**
0.629	0.726	0.000	0.000	0.000	0.039	97.953	0.653	0.000	0.000	0.000

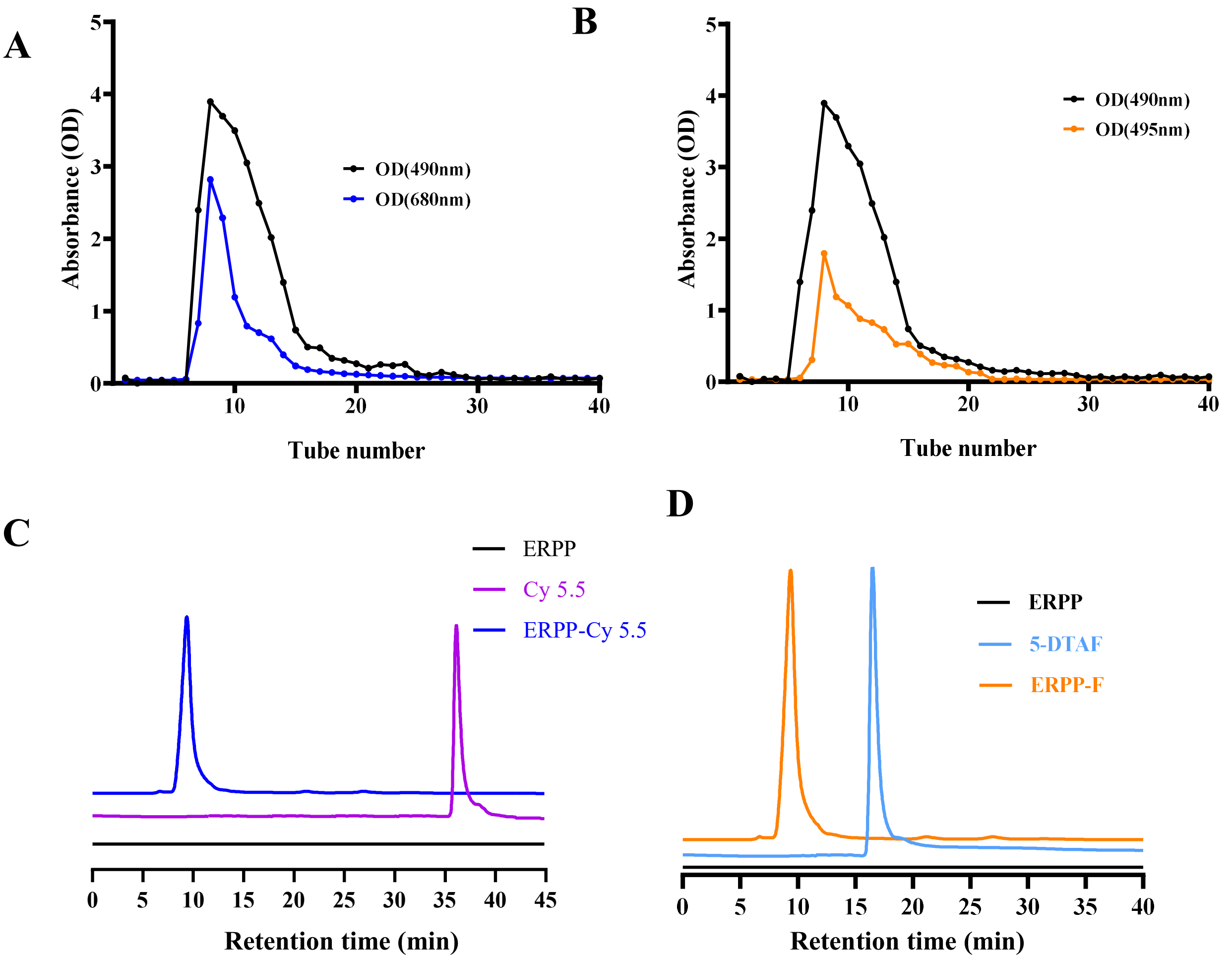

Fig. S2. Chromatographic and spectroscopic evidence for covalent conjugation and purification of fluorescently labeled ERPP. (A) Sephadex G-25 column elution profile of ERPP-Cy5.5 monitored simultaneously at OD 490 nm and OD 680 nm. (B) Sephadex G-25 column elution profile of ERPP-Cy5.5 monitored simultaneously at OD 490 nm and OD 495 nm. (C) HPGPC-FLD chromatogram of ERPP, free Cy5.5, and ERPP-Cy5.5. (D) HPGPC-FLD chromatogram of ERPP, free 5-DTAF, and ERPP-F. The extended retention time of free Cy5.5 relative to free 5-DTAF under identical SEC conditions is attributable to non-specific hydrophobic interactions between the lipophilic cyanine fluorophore and the column matrix, a behavior characteristic of cyanine-type dyes; this does not affect the resolution between ERPP-Cy5.5 and free Cy5.5, nor the validity of the purification assessment.

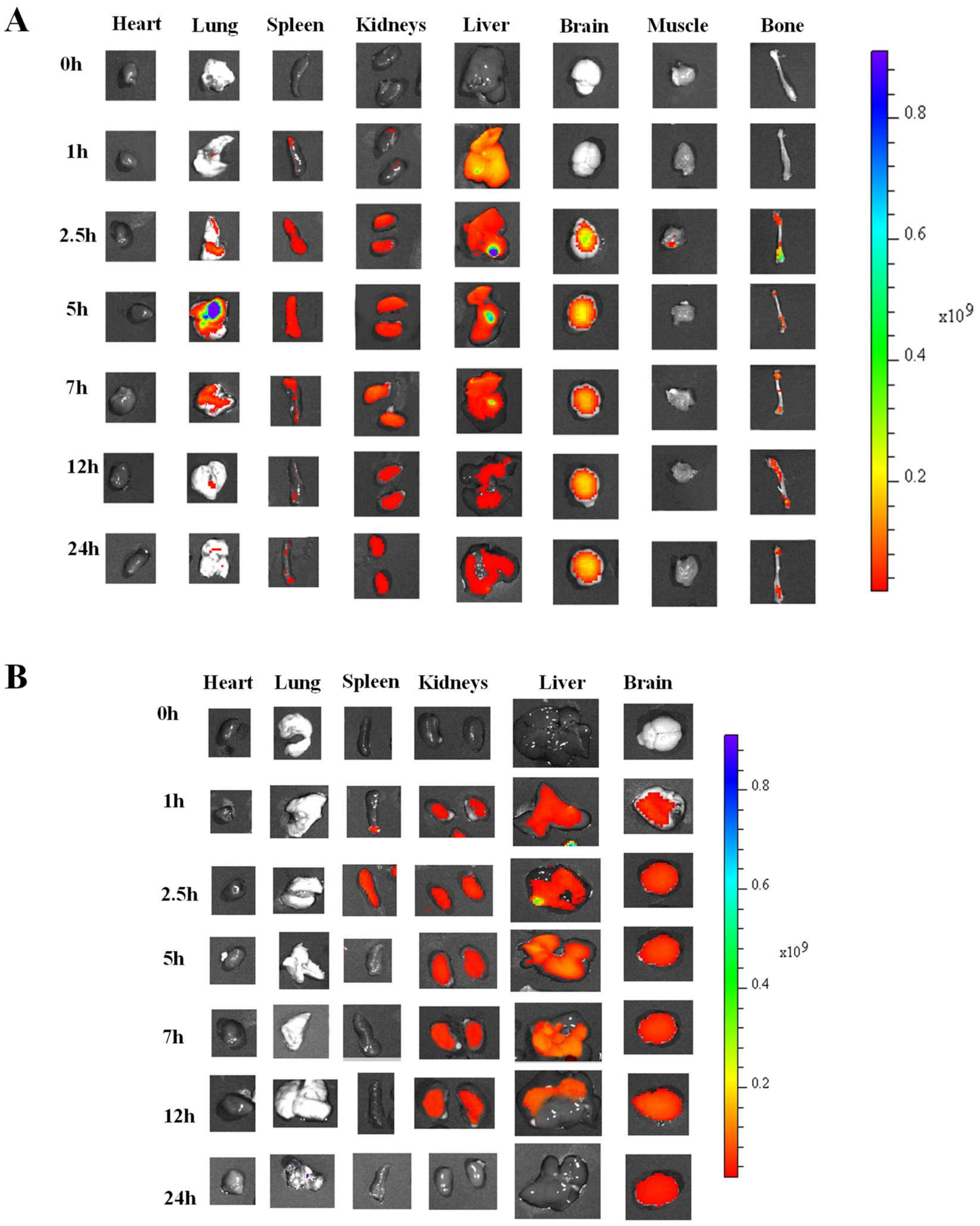

Fig S3. (A) *Ex vivo* imaging of main organs (Heart, Lung, Spleen, Kidneys, Liver, Brain, Muscle, and Bone) after oral administration of ERPP-Cy5.5. (B)) *Ex vivo* imaging of main organs (Heart, Lung, Spleen, Kidneys, Liver and Brain) after oral administration of free Cy5.5.

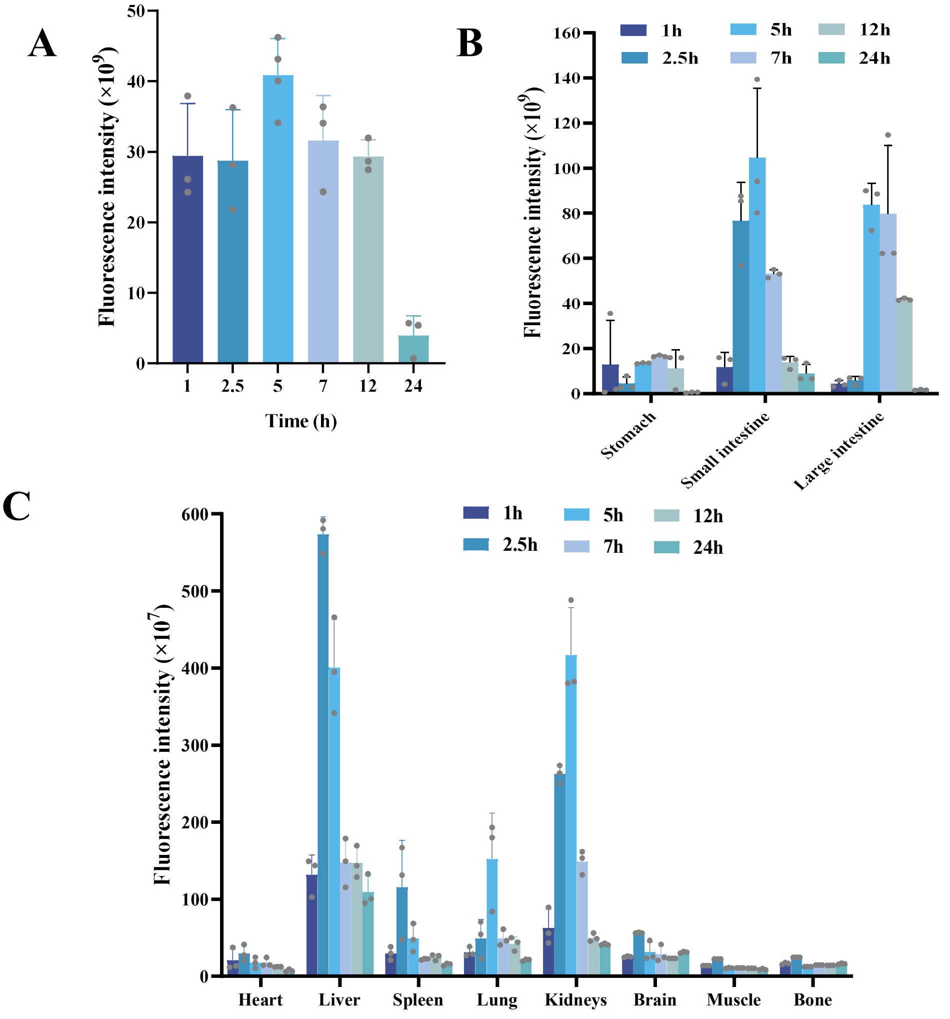

Fig S4. (A) The ROI value of abdomen after oral administration of ERPP-Cy5.5. (B) The ROI value of different gastrointestinal (Stomach, Small intestine and Large intestine) after oral administration of ERPP-Cy5.5. (C) The ROI value of main tissues (Heart, Lung, Spleen, Kidneys, Liver, Brain, Muscle, and Bone) after oral administration of ERPP-Cy5.5.
